# Meningitis Studied by the Ophthalmoscope

**Published:** 1863-10

**Authors:** M. Bouchut


MENINGITIS STUDIED BY THE OPHTHALMOSCOPE—BY M. BOUCHUT.

We have already before called the attention of our readers to the value of the ophthalmoscope for the diagnosis of meningitis and other cerebral affections.

Twenty-three cases of meningitis have been examined in Bouchut's Clinic. The changes in the eye-ground, observed in these cases, were:

1st—Peripheric congestion of the optic nerve and congestive exsudations in the retina and choroid.

2d—Dilatation of the veins of the retina around the papilla.

3d—Varicosity and flexuosity of those veins.

4th—Thrombosis of those veins.

5th—Retinal hæmorrhages in consequence of rupture of veins in some cases.

The papilla is always less distinct; its circumference is diminished in consequence of the surrounding congestion.

In one case, cerebral symptoms had raised the suspicion of meningitis, and the ophthalmoscope showed the characteristic symptoms. The cerebral symptoms subsided entirely, but the child died twelve days afterwards, in consequence of general tuberculosis. The post-mortem showed that the ophthalmoscopic examination had not deceived, for there existed a great number of meningeal tubercles.

M. Robin examined, at the request of M. Bouchut, a number of the eyes microscopically. He found the retinal veins dilated, sometimes containing clots, sometimes ruptured in consequence of hæmorrhages. Once the internal and middle tunic of a vein were broken, and the vein dilated at that point presented a kind of aneurism. In one case, the papilla was irregular; another time, white patches, looking as if the tissues had undergone fatty degeneration, appeared; in three cases, at an examination shortly before death, a decoloration of the eye-ground led to suppose an anæmic condition, while previously congestion had been found.

The congestion of the deep membranes of the eyes Mr. B. explains in the following way: The veins of the choroid and retina, he says, issue into the *sinus cavernosi*, and as soon as the flow of blood in the different sinuses of the *dura mater* is impeded, the circulation of the veins of the deep coats of the eye must be influenced correspondingly by it, and necessarily the circulation in the sinuses of the membranes of the brain is rendered more difficult in meningitis, either by the great congestion, or thrombosis of the various intra-cranial sinuses.

Often the degree of congestion shown by the ophthalmoscope is different in both eyes, as the degree of intra-cranial venous circulation is not equal on both sides.

The deformity of the papilla may sometimes, the author says, owe its existence to the compression of the optic nerves near the chiasma by purulent or gelatinous exsudations, as they are frequently observed in tubercular meningitis.

M. Bouchut, one of the first medical authorities in France, considers the ophthalmoscope as a valuable and almost indispensable means of the diagnosis of cerebral diseases, particularly in children, and we hope that our readers will have an opportunity to try and give us the result.—*Gazette des Hop., Oct.* 1862,—*Am. Jour. Ophthalmology.*

